# From the drama of unoccupied time and isolation due to Covid-19’s pandemic to the need for person-centered care at residential care facilities in Portugal

**DOI:** 10.1007/s12144-022-03499-9

**Published:** 2022-08-05

**Authors:** Maria Miguel Barbosa, Constança Paúl, Laetitia Teixeira, Javier Yanguas, Rosa Marina Afonso

**Affiliations:** 1 Health Sciences Research Centre of the University of Beira Interior (CICS-UBI) - Faculdade de Ciências da Saúde, Av. Infante D. Henrique, 6201-506 Covilhã, Portugal; 2 CINTESIS@RISE, ICBAS, University of Porto, Rua Dr. Plácido da Costa, s/n, 4200-450 Porto, Portugal; 3 Instituto de Ciências Biomédicas Abel Salazar, School of Medicine and Biomedical Sciences, University of Porto, Rua de Jorge Viterbo Ferreira, 228, 4050-313 Porto, Portugal; 4 Aubixa Fundazioa, San Sebastián, Spain; 5 Faculdade de Ciências Sociais e Humanas, University of Beira Interior, Estrada do Sineiro, s/n, 6200-209 Covilhã, Portugal

**Keywords:** COVID-19 pandemic, Older adults, Person-centered care, Residential care facilities, Occupation activities, Social Isolation

## Abstract

During the pandemic, restrictive measures were implemented at Portuguese residential care facilities (PRCF), such as isolating residents and ceasing collective activities. It is important to understand how PRCF are implementing activities that allow residents to occupy their time and fight isolation. As such, we aim to analyze whether: 1. new activities were implemented for residents (identifying which were carried out); 2. occupation activities were provided to isolated residents in their rooms (identifying which were carried out); 3. the implementation of activities is associated with variables like the amount of staff. This is an exploratory, quantitative, and cross-sectional study. An online questionnaire was sent by email to 2325 PRCF and entities were asked to share it with their workers. The study was also divulged on social networks. Data collection occurred between July 8th and October 18th, 2020. The study had 784 staff members participating and 90.8% reported that new activities were implemented at their facilities, predominantly videocalls. Concerning isolated residents most respondents (64.4%) stated that providing activities was impossible. Results showed that those PRCF that expanded teams had a higher percentage of new activities and activities with residents isolated in bedrooms. These results are alarming because while residents should have had more resources to cope with the pandemic, higher risks of unoccupied time and isolation existed, a dramatic situation for its potentially harmful consequences. Focusing on sanitary issues (and less on older adults) may reinforce traditional care models that had shown negative impacts before the pandemic. This highlights the need to evolve the care paradigm during and beyond the pandemic at PRCF: with Person-Centered Care as an option.

## Introduction

Portugal is the third country with the highest aging rate in Europe (PORDATA, 2018) and there are more than 2500 certified residential care facilities with about 100,000 residents (Ministry of Labor, Solidarity and Social Security, 2020). These structures are managed by technical directors and allow for the collective housing of Older Adults (OA), with different degrees of dependency, with temporary or permanent use, and provide services related to food, hygiene, support in the performance of daily living activities, health care and ludic/occupation activities (Ministry of Labor, Solidarity & Social Security, 2012).

Ludic/occupational activities consist of diversified dynamic processes (individual or in groups) of participation, which should be significant to the person, maintain a certain structure and infer the stimulation of various dimensions. The participation of OA in ludic/occupation activities promoted at residential care facilities has proven benefits as it allows them to occupy and structure their time where they live, allows them to fight isolation, facilitates personal development, and optimizes physical, cognitive, emotional and social functionality (Guimarães et al., 2016; Martínez et al., 2015; Morley et al., 2014; Sepúlveda-Loyola et al., 2020). It is noteworthy that these activities provide an opportunity for enjoyment, affective interaction/coexistence and to fight boredom and loneliness, reducing the impact of stressful factors and symptoms of anxiety and anguish (Molony et al., 2018; Monahan, et al., 2020; Simard & Volicer, 2020; Steward, 2020; Stewart et al., 2017). Engaging in meaningful, pleasant, and rewarding activities promotes a growth in well-being and quality of life (Cermak, & Borreson, 2016; Martínez, 2016; Morley et al., 2014; Stewart et al., 2017), thus becoming a very relevant element in terms of promoting the physical and mental health of residents of residential care facilities.

The residents of residential care facilities are heavily impacted by the pandemic of the Coronavirus disease (Covid-19)—declared in March 2020 by the World Health Organization (Direção Geral da Saúde—DGS, 2020)—and represent about half of the deaths due to this disease in several European countries (Inzitari et al., 2020; Sands et al., 2020). Despite heterogeneities between countries, the Covid-19 pandemic showed a tendency to exacerbate pre-existing vulnerabilities in residential care facilities (e.g., infrastructure, work organization, low number of staff members, who are also poorly skilled and poorly paid), creating environments with low resilience to critical circumstances (Ayalon et al., 2020; Inzitari et al., 2020; Lemire, 2020; Pitkälä, 2020; United Nations, 2020). Not only are people living at residential care facilities particularly vulnerable (e.g., dependency, comorbidities), there’s also a high risk of transmission as there are often agglomerations of residents living in confined environments (Dichter et al., 2020; Gardner et al., 2020). As a result, a robust infection prevention/control program has been implemented to protect residents and staff (WHO, 2020), with residential care facilities being the target of extremely restrictive measures around the world (Verbeek et al., 2020). In Portugal, some of the guidelines and measures for the prevention and control of Covid-19 infection at residential care facilities included: a) physical distance between people; b) decrease in the circulation of residents inside the institution (e.g., keeping them isolated in their bedrooms); c) shift-based access to common areas; d) compliance with an isolation period of no less than 14 days in the case of users who exited the institution; e) suspension of visits (in March 2020, resumed on May 2020, with mandatory use of masks and physical distancing, as well as scheduled and limited time for visits in specific and controlled spaces); f) reorganization of daily activities; and g) cessation of collective ludic activities (DGS, 2020; Portuguese Government, 2020).

Therefore, according to these pandemic mitigation measures, OA residing at Portuguese residential care facilities found themselves restrained to the institutions, restricted from circulating freely through the facilities, isolated in their bedrooms, without visits (or visits with distancing and no touching), with their daily activities being reorganized and their collective ludic activities terminated. These factors lead to the risk of changing daily routines, limiting daily experiences, reducing the opportunity to enjoy the benefits of participating in activities, a lack of stimulation and personal interaction, which can enhance isolation and unoccupied time. Several studies report on the negative effects that the mitigation measures had on OA, on a physical and mental health level (e.g., Dichter et al., 2020; Levi-Belz & Aisenberg, 2020; Moro & Paoli, 2020; Narici et al., 2021; Sepúlveda-Loyola et al., 2020; United Nations, 2020; Van der Roest et al., 2020; Wu, 2020).

Residential care facilities in Portugal assume a central role in protecting the physical and mental conditions of OA (Gil, 2019a; Ministry of Labor, Solidarity and Social Security, 2020), but while the participation of OA in ludic/occupation activities has several benefits (Guimarães et al., 2016; Martínez et al., 2015; Morley et al., 2014; Sepúlveda-Loyola et al., 2020), residential care facilities had to adapt their dynamics to serve sanitary criteria and mitigation measures related to the pandemic that forced them to cease and adapt activities which could have psychosocial risks and compromise the well-being and quality of life of residents (Dichter et al., 2020). Considering these circumstances, it is necessary to describe and critically reflect on the current situation in order to support technical, political and scientific advancements to deal with the pandemic crisis and protect the well-being, the quality of life and the dignity of residents at residential care facilities.Therefore, it is important to understand how the Portuguese residential care facilities are implementing activities that allow residents to occupy their time and fight isolation. Using the insight of staff from the Portuguese residential care facilities, the present study has the following specific objectives: 1) to analyze whether new activities were implemented for the residents and 1.1.) identify which activities were carried out; 2) to assess whether occupation activities were provided to residents isolated in their bedrooms and 2.1.) identify which activities were carried out; and 3) to analyze whether the implementation of activities (i.e. new activities and occupation activities for residents isolated in their bedrooms) is associated with the existence of Covid-19 cases in the institution, the type of residential care facilities and the number of staff members.

## Materials and methods

### Study design and ethics

This quantitative and cross-sectional research is part of the project: “Atenção Centrada na pessoa na prestação de cuidados na velhice: abordagens e instrumentos de avaliação” approved by the Ethics Committee from the Universidade da Beira Interior (CE-UBI-Pj-2019-057- ID1555) with an addendum authorized on July 21st, 2020 for the study “Pandemia Covid-19 e cuidados em Estruturas Residenciais para Pessoas Idosas”.

### Procedures and protocol

The study addressed the staff members employed at Portuguese residential care facilities. Participants’ inclusion criteria included having worked at the organization during the first lockdown (March/April 2020), having worked at the organization since at least January 2020 (3 months before the pandemic), and accepting the commitment to participate in the study. No exclusion criteria were considered.

In order to promote a global data collection, all of the residential care facilities in mainland Portugal (*N* = 2250) and the Islands (*N* = 75) were contacted. The contact database included: all contacts present in the Carta Social (the official information on social services in operation in mainland Portugal—Office of Strategy and Planning of the Ministry of Labor and Social Security of the Portuguese Republic) and all contacts obtained through the Social Security Institutes (for the Azores and Madeira) and/or through the official online pages of social institutions (available in Google Maps listing). This process comprised sending 2325 emails. In addition, the researchers used the social networks pages (namely Facebook) of their universities, research units, and personal, to disseminate the study and involve more participants. The contact with residential care facilities included a description of the project, objectives, the link to access the questionnaire in Google Forms, and a request to share the link with the institutions’ staff members (meaning that there could be several responses per institution). The link contained an informed consent with the context of the study, objectives, voluntariness, guarantee of confidentiality and the availability of an investigation team member for contact and clarification.

The protocol was divided into three parts: sociodemographic and professional component, residential care facilities characterization, and two Yes or No questions regarding the implementation of activities with residents during the pandemic: “During the pandemic, were new activities/practices implemented for the users?” and “Was it possible to provide occupation activities to users who were obliged to remain in isolation in their rooms?”. If the participants answered Yes to any of these questions, they were requested to specify the activities performed. The online data collection was carried out between July 8th and October 18th, 2020.

### Data analysis

Regarding the exploratory nature of the study, a descriptive analysis of sociodemographic and professional characteristics of participants was performed through measures of central tendency (mean or median) and dispersion (standard deviation [sd] or interquartile range [IQR]) or absolute and relative frequencies. Additionally, the frequencies of new activities and also activities with residents isolated in their bedrooms were obtained. Chi-Square tests were performed to evaluate the association between activities (i.e. new activities and occupation activities for residents isolated in their bedrooms) and the existence of Covid-19 cases in the institution, the type of residential care facilities and the number of staff members. Statistical analysis was performed using IBM SPSS Statistics 26 and a significance level of 0.05 was considered.

### Participants

The characteristics of the study sample (N = 784) are shown in Table 1. Participants’ ages vary between 19 and 71 years of age (mean = 38.19 years, sd = 9.11 years) and almost all are women (92.7%, *n* = 727). In terms of educational level, 82.1% (n = 644) of the sample had more than 13 years of formal education. The main respondents of the questionnaire were technical directors (41.6%, *n* = 326), direct care providers (17.1%, *n* = 134) and nurses (10.5%, *n* = 82) and the median time of work in the area of OA care was 108.00 months (IQR = 129.00 months). The majority of staff members (79.5%, *n* = 623) reported working in Private Institutions of Social Solidarity (non-profit organizations formed exclusively through the initiative of entities), and the number of users ranged between 4 and 154 users. Responses were obtained from staff members working at institutions from all health regions of mainland Portugal and the islands (according to the division adopted by the DGS).

**Table 1:** Sociodemographic and professional characteristics of participants (*N* = 784) and the characterization of residential care facilities

Sociodemographic and professional characteristics of participants	*n*	*n* (%)/Mean (sd)/median (IQR)
Age [range: 19–71]	768	M = 38.19 (sd = 9.11)
Gender	780	
Female		727 (92.7%)
Male		53 (6.8%)
School Education	780	
Between 1 and 4 years		4 (0.5)
Between 5 and 7 years		2 (0.3)
Between 8 and 9 years		31 (4.0)
Between 10 and 12 years		99 (12.6)
More than 13 years		644 (82.1)
Occupation	783	
Technical director		326 (41.6%)
Direct care provider		134 (17.1%)
Nurse		82 (10.5%)
Entertainment coordinator		68 (8.7%)
Social worker		33 (4.2%)
Psychologist		30 (3.8%)
Others		110 (14%)
Months of work in the area of OA care [range: 7–480]	784	Mdn = 108.00; IQR = 129.00;
Type of residential care facilities management	769	
Private Institution of Social Solidarity		623 (79.5)
Private (company)		146 (18.6)
Number of users [range: 4–154]	726	Mdn = 50; IQR = 38
Geographical area	781	
North		193 (24.6%)
Centre		307 (39.2%)
Lisbon and Tagus Valley		154 (19.6%)
Alentejo		49 (6.3%)
Algarve		27 (3.4%)
Azores		22 (2.8%)
Madeira		29 (3.7%)

Comparing the number of staff members providing direct care before and during the pandemic at the institutions where participants worked, 51.4% (*n* = 403) of the sample indicated that this number was maintained, 28.4% (*n* = 223) of the sample reported that there were fewer staff members providing direct care during the pandemic and 19.8% (*n* = 155) reported that the number of staff members increased.

## Results

### Implementation of new activities with residents during the pandemic

Approximately 91% (*n* = 712) of respondents reported that new activities were implemented for residents, mostly videocalls (76.3%, *n* = 598) (Table 2).

**Table 2:** New activities implemented for residents and its association with the existence of Covid-19 cases, type of residential care facility and number of staff members providing care during the pandemic

New activities	*n* (%)		
Videocalls	598 (76.3%)		
Visitation through physical barrier (e.g., glass, drive thru)	63 (8%)		
Reinforcement of entertainment/occupation activities	45 (5.7%)		
Phone calls	33 (4.2%)		
Reinforcement of individual activities	27 (3.4%)		
Reinforcement of physical activity/physiotherapy	19 (2.4%)		
Sharing photos/videos online	16 (2%)		
Outdoor activities	13 (1.7%)		
Sending photos/videos to family members	13 (1.7%)		
Cognitive stimulation	10 (1.3%)		
Sending letters	7 (0.9%)		
Psychological counseling/psychosocial support	6 (0.8%)		
Others	45 (5.7%)		
	Carrying out new activities		
	Yes *n* (%)	No *n* (%)	*p*
Covid-19 cases at residential care facilities:			
Existence	130 (91.5%)	12 (8.5%)	.813
Non-existence	581 (90.9%)	58 (9.1%)	
Type of residential care facilities:			
Private Social Solidarity Institution	568 (91.5%)	53 (8.5%)	.886
Private (company)	133 (91.1%)	13 (8.9%)	
Number of staff members providing care during the pandemic:			
Maintained	366 (91.3%)	35 (8.7%)	.014
Increased	149 (96.1%)	6 (3.9%)	
Decreased	195 (87.4%)	28 (12.6%)	

There is a statistically significant association between carrying out new activities and the number of staff members providing care during the pandemic. Those residential care facilities that increased the number of staff members providing care had a higher percentage of carrying out new activities (96.1%, *n* = 149) than those that decreased (87.4%, *n* = 195) or maintained (91.3%, *n* = 366) the number of staff members (Table 2). There is no statistically significant association between undertaking new activities and the existence or non-existence of Covid-19 cases and the type of residential care facilities (i.e., whether they are Private Social Solidarity Institutions or private institutions/company) (Table 2).

### Implementation of activities with residents isolated in their bedrooms

Regarding residents who remained isolated in their bedrooms, most staff members (64.4%, *n* = 505) reported that it was not possible to provide activities that allowed them to occupy their time. In cases where it was possible (34.7%, *n* = 272), the most dynamic activities were entertainment (20.2%, *n* = 158) and cognitive stimulation (7.5%, *n* = 59) (Table 3).

**Table 3:** Activities carried out with residents isolated in their bedrooms and its association with the existence of Covid-19 cases at the institution, the type of residential care facilities, and the number of staff members providing care during the pandemic

Activities:			*n* (%)
Entertainment activities (e.g., artistic activities, playful games)			158 (20.2%)
Cognitive stimulation			59 (7.5%)
Physical activity/physiotherapy			52 (6.6%)
Reading (e.g., newspapers, magazines)			45 (5.7%)
TV/radio			30 (3.8%)
Videocalls			19 (2.4%)
Carrying out activities that had been implemented before the pandemic			11 (1.4%)
Psychological counseling/psychosocial support			10 (1.3%)
Films			7 (0.9%)
Use of new technologies (e.g., tablet, platforms and virtual games)			7 (0.9%)
Music therapy			7 (0.9%)
Sensory/Snoezelen stimulation			7 (0.9%)
Religious activities			6 (0.8%)
Conversations through a window/door			4 (0.5%)
Others			22 (2.8%)
	Carrying out activities with residents isolated in their bedrooms		
	Yes *n* (%)	No *n* (%)	*p*
Covid-19 cases at residential care facilities:			
Existence	53 (37.3%)	89 (62.7%)	.522
Non-existence	219 (34.5%%)	416 (65.5%)	
Type of residential care facilities:			
Private Social Solidarity Institution	201 (32.5%)	417 (67.5%)	.004
Private (company)	65 (45.1%)	79 (54.9%)	
Number of staff members providing care during the pandemic:			
Maintained	130 (32.7%)	268 (67.3%)	.000
Increased	75 (48.7%)	79 (51.3%)	
Decreased	65 (29.3%)	157 (70.7%)	

There is a statistically significant association between carrying out occupation activities with residents during isolation in their bedrooms and the type of residential care facilities. Specifically, the private residential care facilities had a higher percentage of carrying out activities with these residents (45.1%, *n* = 65) than the Private Social Solidarity Institutions (32.5%, *n* = 201). There is also a statistically significant association between the implementation of activities with residents isolated in their bedrooms and the number of staff members providing care during the pandemic. The residential care facilities that increased the number of staff members had a higher percentage of activities with these residents (48.7%, *n* = 75) than those that decreased (29.3%, *n* = 65) or maintained (32.7%, *n* = 130) the number of staff members (Table 3). There is no statistically significant association between carrying out occupation activities with residents isolated in their bedrooms and the existence or non-existence of Covid-19 cases at residential care facilities (Table 3).

## Discussion

This study aimed to explore how the Portuguese residential care facilities implemented activities to occupy the time and fight the isolation of its residents. With regard to the first specific objective of this study, which was analyze whether new activities were implemented for residents and identify which activities were carried out, the results show that new activities were implemented for residents. However, the new activities were essentially videocalls, and considering their frequency (> 70%), this practice seems to have been a panacea, that is, a transversal solution that residential care facilities found to fill the gap left by the isolation and a lack of visitation. These results are coherent with the study by Naudé et al. (2022), which shows that many geriatric institutions around the world promoted videocalls due to the pandemic. Although videocalls can provide a solution regarding connection and social contact at a distance (Inzitari et al., 2020), they tend to be sporadic and short-lived activities (Naudé et al., 2022). Additionally, several technical and human factors can become barriers in the use of videocalls, such as the cognitive and physical capacities required, the complexity of technological devices and the limited digital skills (Naudé et al., 2022). According with the study by Gil (2019b), Portuguese OA have low digital literacy. For these cases, authors Ayalon et al. (2020), Trabucchi and De Leo (2020), emphasize in their studies about the pandemic at residential care facilities that the use of technological means has limited effectiveness and sometimes causes serious discomfort, meaning that conducting videocalls to alleviate loneliness and anxiety may have an opposite effect to the one intended. Consequently, in cases where OA cannot use new technologies on their own, they will be excluded or will require help from staff members (Naudé et al., 2022; Seifert et al., 2020; United Nations, 2020). This raises ethical considerations related to privacy (Naudé et al., 2022). If fully provided and maintained throughout the videocall, the help and support provided by staff members can jeopardize the privacy of interactions between OA and their relatives. Therefore, and even when well intentioned, it may constitute a violation of the right to reserve the intimacy of private life according to the 26^th^ article of the Portuguese Republic Constitution (2004).

Recommendations from several authors and entities emphasize the importance of preventing any regression of abilities, as well as physical and mental health problems in OA during the pandemic crisis (Dichter et al., 2020; Sepúlveda-Loyola et al., 2020; United Nations, 2020; Wu, 2020). At this level, maintaining previously existing activities related to the physical component, as well as the existence of cognitive stimulation practices, outdoor activities and psychological counseling/psychosocial support, has shown a concerning small expression in our study’s findings. Moreover, as residents cannot participate in group activities (DGS, 2020; Portuguese Government, 2020), which tend to be standardized, performing individual dynamics could impose an opportunity to develop personalized and meaningful activities, something that could be beneficial in the current pandemic context. However, results have shown a residual expression concerning the reinforcement of individual activities (3.4%). Regarding the second objective of this study, which was to assess whether occupation activities were provided to residents isolated in their bedrooms, most participants (> 60%) reported that it was not possible to provide activities. It is the authors’ opinion that this alarming result is associated with a double risk of exclusion, since these OA are not only confined to a restricted space, but also lack activities that provide them with stimulation, interactions and occupy their time. This situation makes it impossible for them to enjoy the benefits of being involved in activities, and increase the feeling of isolation and unoccupied time, which according to Abbasi (2020), Haj et al. (2020) and Wu (2020) are threats caused by the pandemic context that have dramatic consequences associated with the deterioration of the physical and mental health state of OA living at residential care facilities. In this regard, there are noteworthy studies (e.g., Abbasi, 2020; Brooke & Jackson, 2020; Chu et al., 2020; Haj et al., 2020; Pitkälä, 2020; Simard & Volicer, 2020), that alert to the risks and consequences at an emotional level (e.g., loneliness, anxiety, depression, desperation), at a cognitive level (e.g., decline, worsening of dementia), at a physical level (e.g., sedentary lifestyle, development of sarcopenia, increased risk of cardiovascular diseases), and an increase in dependency and mortality from causes unrelated to the Covid-19 disease, but under its critical context. In other words, the impact of the pandemic on these residents, whose situation of confinement in a restricted space should have been compensated for, increased and posed risks with potentially dramatic results, since residential care facilities were not able to provide occupation activities.

In cases where activities were provided to residents isolated in their bedrooms, this study would make note of entertainment activities, physical and cognitive stimulation, activities already implemented before the pandemic, as well as psychological counseling/psychosocial support. If the duration, quantity and quality of interventions is adequate, these activities may prevent the deterioration of physical and mental abilities during the pandemic crisis (Dichter et al., 2020; Sepúlveda-Loyola et al., 2020; United Nations, 2020; Wu, 2020). Activities like watching television and listening to the radio are also noteworthy since they are in line with what Portuguese OA tendentially choose to occupy their time (Martins, 2010). Alarmingly, communication/contact activities had low expression among the participants responses, which may suggest the lack of opportunities for OA to communicate with relatives/outsiders, resulting in an aggravated isolation.

Concerning the third objective, which was to analyze whether the implementation of activities was associated with other variables, results have shown that those residential care facilities that increased the number of staff members providing care not only had a higher percentage of new activities but also a higher percentage of activities with residents isolated in their bedrooms. These results seem to suggest that a higher staff level at residential care facilities leads to a better ability to respond to the pandemic crisis in terms of implementing occupation activities and fighting isolation. This means that, while the institutions with an increased staff level probably had more human resources to support the OA, residential care facilities that did not increase the number of staff members probably were less prepared to respond to these critical circumstances regarding the occupation of resident’s time and fighting their isolation. This result is consistent with the literature that reveals that a low number of staff members fosters the low resilience of residential care facilities to critical circumstances (Ayalon et al., 2020; Inzitari et al., 2020; Lemire, 2020; Pitkälä, 2020; United Nations, 2020), and that increasing the proportion of caregivers facilitates the quality of care (Barbosa et al., 2020; Castle & Engberg, 2007). This leads to considerations on the importance of human resources and the need to increase them at Portuguese residential care facilities. Lastly, both the implementation of new activities and carrying out occupation activities with residents isolated in their bedrooms were not associated with the existence of Covid-19 cases at residential care facilities. This seems to reflect that the lack of activities in the Portuguese residential care facilities occurred regardless of the existence of outbreaks at the institutions.

This study has limitations namely in regards to the impossibility of calculating the answer rate. This occurs due to the inexistence of official data on the number of employees at residential care facilities and the impossibility of identifying the participating institutions, stemming not only from the assured data confidentiality but also from the possibility of several responses per institution. The data must be contextualized within the specific time interval (after the first wave) in which they were collected. The asymmetrical development of the pandemic in the country means that residential care facilities had to manage the pandemic at different stages, which can influence the results. It is likely that staff members who volunteered to participate in this study were the least overburdened at the time of the research. Collecting data online may have made it difficult for more staff members to participate, especially those with low digital literacy/use, which could indicate that the sample is not representative. However, given the strict standards of infection control that prevented a one-to-one data collection and the use of printed questionnaires at residential care facilities, a remote online data collection was the only viable option.

There’s still plenty to study concerning this pandemic context, and considering that videocalls were the main response presented by residential care facilities to this pandemic crisis in terms of implementing activities, it is important to further study the impact of videocalls on OA, their benefits and downsides. It is equally essential to understand how to enhance the effectiveness of this practice (e.g., increasing the digital literacy of Portuguese OA), as well as how it should be performed (e.g., duration, type of equipment, and accessibility) in order to adapt to the characteristics, needs and rights of OA. Taking into account the alarming result obtained concerning the lack of activities for residents isolated in their bedrooms, future studies are necessary to understand how to fight their isolation and lack of activities, and how to promote the well-being and quality of life of residents at residential care facilities in fruitful ways during and beyond critical situations, such as the current one.

## Conclusion

In Portugal, residential care facilities assume a central role in providing formal care and support to OA. The promotion of ludic/occupation activities should be highlighted for its benefits and relevance in structuring time. The role of residential care facilities became even more prominent during the pandemic since residents depended mainly on what was provided by the facilities as they found themselves confined in their institutions, limited from free circulation, without ludic collective activities and without visitation (or severely adapted). The current pandemic crisis is an unprecedented situation that must be dealt with concomitantly and, considering that the Portuguese residential care facilities were already showing some pre-existing vulnerabilities, it became crucial to produce literature describing the way residential care facilities are dealing/have dealt with such demanding circumstances. Only then is it possible to gather information that may serve as the basis for informed decision-making, to support technical, political and scientific advancements, and to prepare recovery plans and care management models that are sustainable and protect the well-being, the quality of life and the dignity of residents.

The results of this study are deeply alarming. Although the results point to the implementation of new activities for residents, these were mainly videocalls, which tend to occupy very little time, are not likely to be applied efficiently and privately with all OA, and have the potential to promote exclusion and expose the OA to the risk of rights violation. The results also indicate that in the majority of cases it was not possible to provide activities for residents isolated in their bedrooms, who required a reinforcement of options for occupying time and fighting isolation. While a better structure of time and access to strategies and resources to deal with the pandemic should have been provided, it is concluded that they were at risk of greater isolation and a lack of occupation activities, a dramatic situation due to its potentially harmful consequences.

The overall results of this study, within the Portuguese context, lead to reflections concerning the negative impact of the pandemic on OA living at residential care facilities, about the emphasis onquantity of life (at the expense of its quality), about loneliness, about digital illiteracy, social segmentation as well as the exposure to the risks of human rights violation. These are potential silent threats that can trigger a hidden pandemic with serious long-term effects. With pandemic-related measures, namely those related to ceasing/adapting activities, the maintenance of basic physical, mental and social abilities, which directly affect the quality of life and dignity of residents, is at risk. Therefore, similarly to the infection control measures, urgent measures are needed to promote meaningful, pleasant and rewarding activities at residential care facilities, allowing its residents to structure their time and fight isolation. The results of this study, related to the association between the amount of human resources and performing activities suggests the relevance of promoting resources at residential care facilities, namely by increasing the number of staff members and by developing their training on infectious-related issues and on promoting the mental health of the people they care for.

Overall, the results seem to reflect the worsening of pre-pandemic vulnerabilities at the Portuguese residential care facilities, namely concerning the practice of a traditional care approach. The traditional care approach tends to focus on the illness and the service, and sees OA as a homogeneous group that passively receives care. This perspective fosters paternalism, standardized procedures, and task efficiency. With the pandemic, the focus on Covid-19 prevention (and less on OA as a whole) accentuates and legitimizes more standardized care practices that may reinforce the traditional care model that had shown signs of exhaustion and negative impacts on OA prior to the pandemic. Therefore, this study emphasizes the urgent need for an evolution of the care paradigm at Portuguese residential care facilities. Person-Centered Care is consequently proposed as an option for being currently recognized as the highest standard of quality in the care of OA (with positive repercussions on the well-being of both residents and caregivers).

Person-Centered Care is based on respect for the rights and dignity of people in need of care, placing them at the center of attention. This approach privileges the person's role as an active decision-maker on the matters of their daily lives, respecting their psychosocial needs and potentialities. This also includes the opportunity to participate in meaningful activities that respect self-determination, individual risk acceptance, depends on the interests/desires of OA, and promotes their personal autonomy. Considering these principles, the pandemic crisis shows an even higher need for person-centered care in order to balance the risks and weigh the need for infection management against the respect for the rights, physical health, needs and well-being of OA, to overcome this crisis with the least possible impact while preserving their dignity and quality of life.

While the pandemic context represents a threat due to the very serious (and long-term) consequences it causes to OA living at residential care facilities, it also forces these organizations to adapt. Therefore, the current crisis is an opportunity for change, as it creates a chance to reflect on quality care and the living conditions of residents at residential care facilities. It is therefore necessary to profoundly redefine priorities and scientifically and politically invest in Person-Centered Care during – and beyond – the pandemic.

## Data Availability

The datasets generated during the current study are not publicly available (since the data subjects were asked to consent to the processing of data only within the scope of the specific objectives of this investigation) but are available from the corresponding author on reasonable request.
